# Benign pancreatic lesion on ^18^F-FDG PET-MRI: A case report

**DOI:** 10.1097/MD.0000000000033706

**Published:** 2023-05-12

**Authors:** Yuqiang Xiao, Yong Zha, Jindan Li, Conghui Yang, Long Chen, Ran Xie

**Affiliations:** a Department of PET/CT Center, Yunnan Cancer Hospital, The Third Affiliated Hospital of Kunming Medical University, Cancer Center of Yunnan Province, Xishan District, Kunming, Yunnan, People's Republic of China; b Department of Hepatobiliary and Pancreatic Surgery, Yunnan Cancer Hospital, The Third Affiliated Hospital of Kunming Medical University, Cancer Center of Yunnan Province, Xishan District, Kunming, Yunnan, People's Republic of China.

**Keywords:** ^18^F-FDG, no FDG avid, pancreas lesion, PET-MRI

## Abstract

Differentiation of benign and malignant lesions in the head of pancreas is the key. When the mass is small, it is difficult to distinguish via conventional imaging modalities. Positron emission tomography and magnetic resonance imaging (PET/MRI) can distinguish benign and malignant lesions from the perspective of metabolism, which provides a new idea for the diagnosis of pancreatic head mass.

**Patient concerns:**

We report the case of a 44-year-old male patient who underwent an ^18^F-fluorodeoxyglucose PET/MRI for medical examination. The patient did not complain of special discomfort.

**Diagnoses:**

PET-MRI revealed in the head of the pancreas, there is a circular space-occupying lesion without obvious fluorodeoxyglucose accumulation, which tends to be benign based on its MRI and metabolic characteristics.

**Interventions:**

The patient refused further laboratory examination or ultrasound gastroscopy as there is no discomfort.

**Outcomes:**

No special discomfort was found in the patient after 6 months follow-up.

**Lessons:**

If routine examination fails to diagnose benign or malignant pancreatic head occupying, and the patient refuses invasive examination, PET-MRI can be performed for identification.

## 1. Introduction

The widespread use of high-quality abdominal imaging has led to an increasing number of patients being diagnosed with pancreatic cysts. Most lesions were found to be pancreatic cystic neoplasms rather than pseudocysts. Cystic tumors are usually found incidentally and are small at the time of discovery.^[1]^ At the same time, it is difficult to distinguish many benign pancreatic lesions from some pancreatic cancers by imaging, and unnecessary surgical treatment is adopted, which brings a certain burden to patients. As an advanced imaging method, 18F-FDG positron emission tomography (PET)-computed tomography (CT) examination has shown important value in the diagnosis of many tumors. PET-magnetic resonance imaging (MRI) has more advantages than PET-CT in the evaluation of pancreatic mass.^[2,3]^ At present, there are only a few reports on the PET-MRI features of benign pancreatic masses.

Here, we report the PET-MRI features of a benign pancreatic lesion and review the relevant literature. The patient has provided informed consent for publication of the case, and written informed consent for publication of the details was obtained from the patient and the next of kin.

## 2. Case presentation

**Figure 1.**
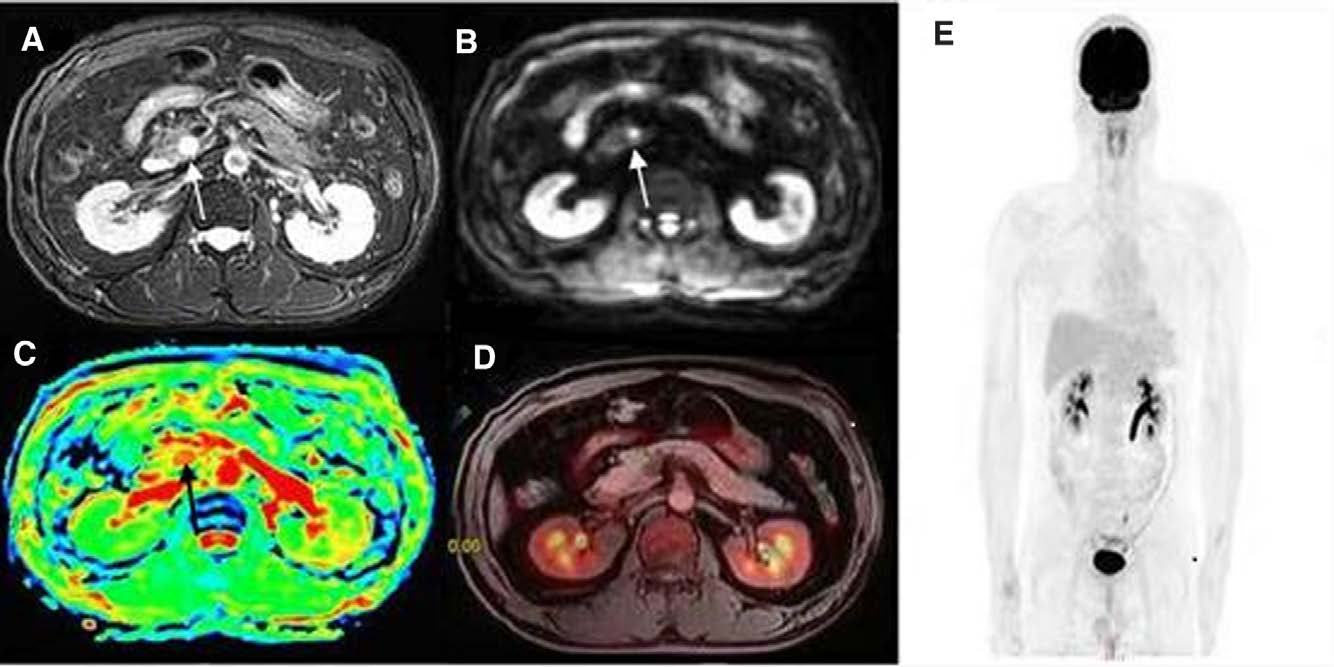
The image of the patient.

A 44-year-old male patient came to our department for medical examination. The patient had no special symptoms. The patient had no family history of related diseases. Physical and laboratory tests showed no abnormalities. T2WI MRI images show a well-defined, round, homogenous high T2 signal mass in the pancreatic head, with no dilatation of the pancreatic duct (Fig. 1A, arrow). DWI demonstrates high signal while ADC shows no obvious restrained difussion (Fig. 1B and C, arrow), indicating a possible benign lesion. Fused Axial PET-MRI showed no significant FDG avid in the mass (Fig. 1D). The maximum intensity projection image (Fig. 1E). Based on the demonstration of ^18^F-FDG PET-MRI, a benign lesion of the pancreatic head was diagnosed. The patient refused further laboratory examination or ultrasound gastroscopy as there is no discomfort. No special discomfort was found in the patient after 6 months of follow-up.

## 3. Discussion

The collecting time of integrated PET-MRI is longer than PET-CT, however, it improves the detection sensitivity of fine lesions.^[2,3]^ Benign pancreatic lesions, which are often found incidentally, are now detected with increasing frequency.^[4]^ Pancreatic masses smaller than 3 cm can not be easily observed by regular image modality, especially in the absence of symptoms.^[1]^ Lesions larger than 3 cm are usually detected by clinical signs and symptoms, elevated pancreatic enzyme concentrations, and abnormal imaging characteristics.^[5]^ According to the 2019 WHO classification criteria for pancreatic tumors, pancreatic tumors were divided into 3 parts: benign epithelial tumors, prodromal tumors, malignant epithelial tumors, and neuroendocrine tumors.^[6]^ The benign epithelial neoplasms and prodromal lesions include serous cystadenoma; pancreatic intraepithelial neoplasia; intraductal papillary mucinous neoplasm; intraductal oncocytic papillary neoplasm; intraductal tubulopapillary neoplasm, and mucinous cystic neoplasm. The annual risk of malignant degeneration of pancreatic cysts is about 0.24%.^[7]^ It varies according to the histological subtype.^[8,9]^ After a pancreatic cyst has been identified on dedicated baseline imaging, subsequent follow-up can be performed by CT or MRI. For patients with nonspecific pancreatic cysts without surgical history, follow-up plans will depend on the patient’s age and cyst size. Follow-up intervals typically ranged from 6 months to 2 years, with a duration of at least 5 to 10 years.^[7–9]^ During follow-up, if “high-risk features” or “worrisome features” are present, immediate endoscopic ultrasonography-guided fine needle aspiration or surgical evaluation should be performed. “High-risk features” included: obstructive jaundice with a cyst located in the head of the pancreas; enhancement of solid components in cysts; the diameter of the main pancreatic duct without obstruction was ≥10 mm. “Worrisome features” include: cyst size > 3 cm; thickening or strengthening of cyst wall; no enhancement of wall tubercles; the diameter of the main pancreatic duct is 5 to 9 mm. In general, invasive carcinoma is less common in asymptomatic cysts (<3 cm).^[10,11]^ Diagnosis of pancreatic masses traditionally relied on the cyst fluid amylase, tumor markers such as CA19-9 and carcinoembryonic antigen. However, the sensitivity and specificity of these markers are largely fluctuant.^[12]^ Further check-ups are needed to differentiate.

In conclusion, benign lesions of the pancreas are relatively common diseases with certain specificity in imaging. In this case, ^18^F-FDG PET-MRI imaging was negative, which provides a new perspective for the diagnosis of small pancreatic head space-occupying lesions. The understanding of space-occupying lesions of the pancreatic head should be strengthened in the work, and the diagnosis should be combined with PET-MRI when necessary, but the final diagnosis still needs pathological evidence.

This study was supported by the National Natural Science Foundation of China (No. 81960496), Yunnan Fundamental Research Projects (No. 202101AT070050), Yunnan health training project of high-level talents (H-2018006). Xing Dian Ying Cai Support Plan.

## Acknowledgments

This study was supported by the National Natural Science Foundation of China (no. 81960496), Yunnan Fundamental Research Projects (no. 202101AT070050, 202101AY070001-157), Yunnan health training project of high-level talents (H-2018006), and Xing Dian Ying Cai Support Plan.

## Author contributions

**Software:** Jindan Li, Conghui Yang.

Writing – original draft: Yuqiang Xiao.

Writing – review & editing: Yong Zha, Ran Xie, Long Chen.
